# Large-scale flood susceptibility mapping along major tributaries of the Nile River system in Sudan using convolutional neural network

**DOI:** 10.1038/s41598-026-58194-7

**Published:** 2026-06-20

**Authors:** Musaab A. A. Mohammed, Abdelrhim Eltijani, Norbert P. Szabó, Péter Szűcs

**Affiliations:** 1 Faculty of Earth and Environmental Sciences and Engineering, University of Miskolc, 3515 Miskolc, Hungary; 2 Department of Hydrogeology, College of Petroleum Geology and Minerals, University of Bahri, Khartoum, Sudan

**Keywords:** Flood susceptibility mapping, Artificial intelligence, Deep learning, Convolutional Neural Network, Nile River system, Flood hazard, Climate sciences, Environmental sciences, Hydrology, Natural hazards

## Abstract

Every fall season, Sudan experiences devastating floods that result in significant fatalities, displacement of populations, and severe disruptions to agricultural and economic activities. The 2020 floods were particularly catastrophic, reaching the highest Nile River levels in over a century and affecting more than 800,000 people. To the best of the authors’ knowledge, this study represents one of the first large-scale, data-driven flood susceptibility assessments for Sudan using deep Convolutional Neural Network (CNN) model. Twelve conditioning factors spanning topographical, hydrological, geological, and anthropogenic parameters were systematically analyzed. The flood inventory dataset was obtained from the Humanitarian Data Exchange (HDX), comprising geospatially verified flood polygons compiled from remote sensing and field observations. A five-fold cross-validation strategy was implemented to ensure model robustness, yielding a classification accuracy of 97% and demonstrating reliable generalizability across spatial partitions. The analysis generated probability maps with susceptibility values ranging from zero to 1, delineating three discrete risk zones: High, Medium, and Low susceptibility. Results reveal that very high susceptibility zones are concentrated along immediate river corridors, the Khartoum metropolitan confluence area, and low-lying Quaternary alluvial plains in Jazirah and Sennar states, where Sudan’s most critical agricultural production occurs. The susceptibility maps provide essential decision-support tools for evidence-based urban planning, agricultural management, and water resources development, enabling targeted risk reduction strategies that can protect lives, livelihoods, and development investments.

## Introduction

Recent global analyses indicate that floods remain the most frequently occurring natural disaster, accounting for over 40% of all disaster events and disproportionately affecting populations in low- and middle-income countries^1^. Their increasing frequency and severity, driven by climate change, rapid urbanization, and poor land-use practices, have intensified the need for effective risk assessment and management^2–5^. Flood susceptibility mapping plays a crucial role in identifying flood-prone areas and supporting evidence-based planning and mitigation. It enables authorities to enforce zoning regulations, design resilient infrastructure, and develop efficient early warning and emergency response systems. Accurate and reliable susceptibility maps are therefore essential tools for reducing flood impacts and promoting sustainable development in vulnerable regions^6^.

Various methodological approaches have been developed for flood susceptibility assessment, each with distinct strengths and limitations. Physics-based hydrological and hydraulic models such as HEC-RAS and MIKE FLOOD simulate rainfall-runoff and flood wave propagation processes to generate detailed inundation maps showing flood depth, velocity, and extent^7–10^. Although these models provide high accuracy, they demand extensive input data and significant computational resources, making them less feasible in data-scarce regions^11^. Statistical approaches use historical flood records to establish empirical relationships between flood occurrence and influencing factors, enabling probabilistic estimation of flood-prone areas^12,13^; however, their reliability depends on the completeness and quality of past data. Multi-criteria decision analysis (MCDA) methods combine multiple conditioning factors through expert-assigned weights^14–17^, offering flexibility and transparency but introducing subjectivity in weighting and threshold selection^18^.

Recent advances in machine learning (ML) and artificial intelligence (AI) have transformed flood mapping by enabling data-driven modeling of complex, non-linear relationships among multiple conditioning factors without relying on explicit physical modeling or subjective expert judgment^19–25^. Algorithms such as Random Forest, Support Vector Machines, Artificial Neural Networks, and ensemble methods have shown superior predictive accuracy compared to traditional techniques^26–28^. These models learn from known flood and non-flood samples to identify key factor combinations that best predict flood-prone areas. At the forefront, deep learning models such as Multi-Layer Perceptron Neural Networks and Convolutional Neural Networks (CNNs) offer advanced capabilities for analyzing spatial data and automatically extracting hierarchical features^29,30^. CNN-based flood mapping delivers highly accurate and spatially detailed assessments, supporting evidence-based flood risk management^31–33^. Despite the proven potential of deep learning in flood modeling globally, no study to date has applied a CNN-based framework to produce spatially explicit flood susceptibility assessment for the Nile River tributary system.

Sudan faces recurrent and devastating seasonal floods, when intense monsoonal rains over the Ethiopian Highlands generate high flows in the Blue Nile, which merges with the White Nile at Khartoum^34^. The central-eastern region hosts most of Sudan’s population, major agricultural areas, and critical infrastructure, making it extremely vulnerable to flooding. Severe flood events in 2019–2021 caused record-breaking Nile levels, affected over 800,000 people, and inundated vast farmlands^35^. This vulnerability stems from a combination of factors, including the highly seasonal hydrology, climate variability, and land-use changes in upstream catchments^36^. Despite the region’s high flood exposure, flood susceptibility assessments remain absent. The urgent need for scientifically robust and spatially explicit flood risk information forms the central motivation for the present research. This study aims to develop a flood susceptibility assessment for the Blue Nile, White Nile, and main Nile River corridors in central-eastern Sudan using CNN deep learning approach integrated with GIS and remote sensing data. This study makes the following scientific contributions: (1) it presents the first large-scale, spatially explicit flood susceptibility assessment for central-eastern Sudan using a deep learning framework, addressing a critical gap given that existing Nile Basin assessments have predominantly focused on downstream reaches or relied on traditional statistical and physics-based models; (2) it demonstrates the applicability of CNN-based patch classification in data-constrained, semi-arid riverine environments; and (3) it advances model transparency through post-hoc permutation feature importance analysis, providing an interpretable link between deep learning predictions and established hydrological principles. Collectively, the results establish a reproducible and transferable deep learning workflow that delivers both practical insights for flood risk management in Sudan and methodological contributions relevant to other data-scarce riverine regions within the Nile Basin and across Africa.

## Materials and methods

### Study area

**Fig. 1:**
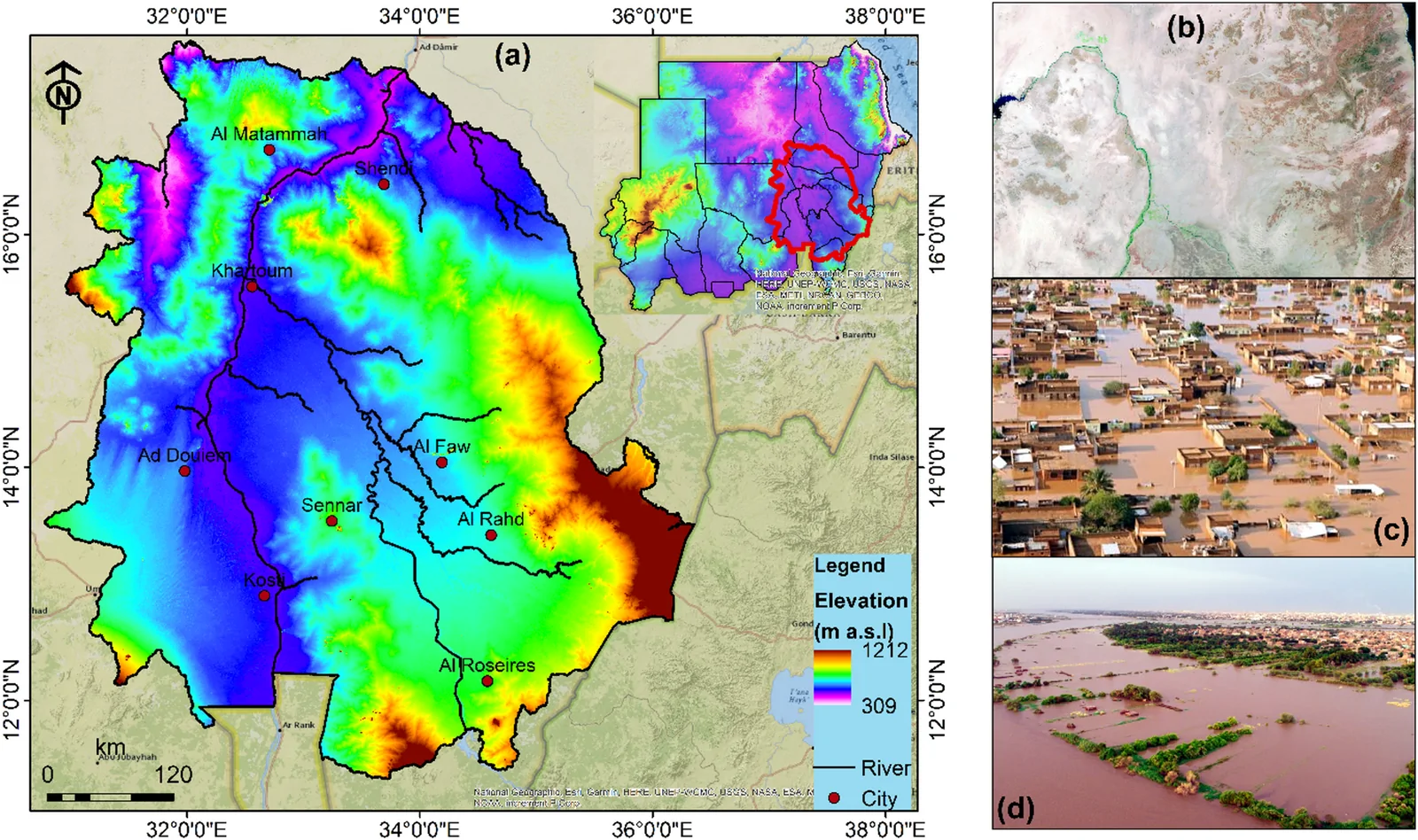
(**a**) Geographical location of the study area in the east-central part of Sudan, (**b**) MODIS-based visual for geographic flood extent in central Sudan, and (**c**) and (**d**) Flooding scenes in Khartoum sourced from Getty Images editorial photos for floods in 2020.

The study area covers the central-eastern region of Sudan along the confluence of the Blue Nile and White Nile, which merge at Khartoum to form the main Nile River (Fig. 1a). The region spans approximately 420,000 km2 and includes River Nile, Khartoum, Kassala, Gedaref, Jazirah, Sennar, and Blue Nile states. The hydrological system is dominated by the Blue Nile, characterized by highly seasonal discharge from the Ethiopian Highlands, and the White Nile, which maintains relatively stable year-round flow^36^. The convergence of these rivers creates extensive floodplains and low-lying alluvial terraces that are naturally prone to inundation.

Topographically, the study area consists mainly of flat to gently sloping alluvial plains, with elevations ranging from about 309 m near Khartoum to over 1200 m in the eastern highlands. The floodplains adjacent to the Blue Nile, White Nile, and main Nile are dominated by Quaternary alluvial deposits that favor riverine flooding and waterlogging (Fig. 1b). The climate ranges from semi-arid to semi-humid, with distinct wet and dry seasons^37^. Annual rainfall shows a strong south-to-north gradient, varying from about 100–200 mm in the northern areas to 400–800 mm in the southern states^38^. The rainy season extends from June to September, coinciding with peak Blue Nile discharge. This seasonal rainfall pattern, combined with monsoonal inflow from the Ethiopian Highlands, is the primary driver of recurrent flooding in the region.

Flood susceptibility is further enhanced by three main geomorphological and hydrological conditions: (1) the highly seasonal Blue Nile discharge, with peak flows during July–September; (2) the extensive low-gradient alluvial plains that limit natural flood attenuation; and (3) the widespread Quaternary deposits in the Jazirah and Sennar plains that are highly susceptible to inundation and waterlogging. These characteristics make the region highly suitable for CNN-based flood susceptibility assessment.

### Database preparation

The development of the flood susceptibility model required integrating multiple spatial datasets representing the physical and anthropogenic drivers of flooding. All data were standardized within a GIS framework (UTM Zone 36N, WGS84) at a 30 m spatial resolution to ensure consistency for pixel-based analysis and model training. Twelve flood conditioning factors were compiled from remote sensing, global datasets, and national sources, categorized into topographical, hydrological, geological, and anthropogenic groups (Table 1). Quality control involved visual inspection, statistical validation, and consistency checks against independent references.

**Table 1:** Datasets used for generating the flood susceptibility map using convolutional neural networks.

Category	Factor	Data source
Topographical	Elevation	SRTM DEM (USGS, 30 m) https://earthexplorer.usgs.gov
	Slope	Derived from DEM
	Curvature	Derived from DEM
	Topographic wetness index (TWI)	Derived from DEM using flow accumulation and slope
Hydrological	Drainage density	Derived from DEM (Flow accumulation analysis)
	Distance to stream	Derived from DEM and stream network
	Stream power index (SPI)	Derived from DEM
	Rainfall	CHIRPS (0.05° resolution) https://www.chc.ucsb.edu/data/chirps
Geological	Lithology	Geological Survey of Sudan
anthropogenic	NDVI	Landsat 8 OLI https://earthexplorer.usgs.gov
	Land cover	ESA Climate Change Initiative (CCI) https://www.esa-landcover-cci.org
	Distance to roads	OpenStreetMap https://www.openstreetmap.org

The flood inventory map, used as the dependent variable in CNN model training, was obtained from the Humanitarian Data Exchange (HDX; https://data.humdata.org), which provides geospatial datasets of recorded flood events in Sudan. The flood inventory covered flood events recorded between 2010 and 2022 and consisted of 47 georeferenced flood polygons. From these polygons, 1,820 flood occurrence points (value = 1) were extracted through systematic GIS sampling. An equal number of non-flood points (value = 0) were generated to maintain a balanced dataset. Non-flood points were sampled exclusively from areas meeting all of the following criteria: elevation greater than the 75th percentile of the study area, TWI values within the lowest quartile, and a distance greater than 2 km from any mapped flood polygon and major watercourse. Although some residual sampling bias may persist, the integration of multiple hydrological exclusion criteria substantially reduces this risk compared with purely random non-flood sampling^28^. To further reduce spatial autocorrelation, a minimum inter-sample distance of 500 m was enforced across both flood and non-flood classes.

### Methods

**Fig. 2:**
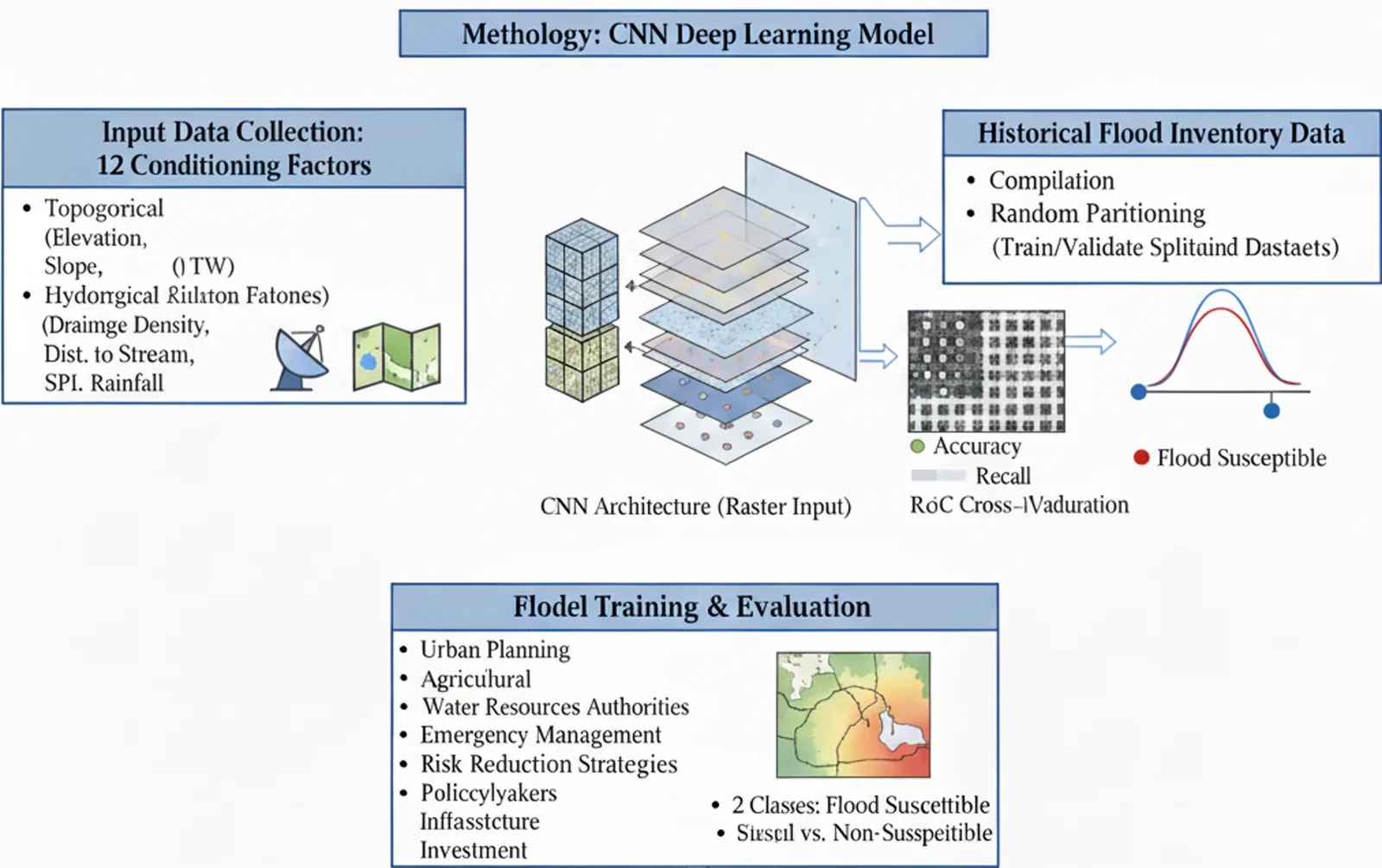
The workflow of the study.

This workflow outlines a flood susceptibility mapping using a Convolutional Neural Network (CNN) deep learning (Fig. 2). It begins with collecting various conditioning factors (topographical, hydrological, and anthropogenic) and historical flood data. All geospatial data preprocessing, including raster alignment, resampling, and spatial overlay operations, was performed in QGIS 3.32 and the Python GeoPandas library. The CNN model was developed and trained using TensorFlow 2.12 and Keras running on Python 3.10. All continuous conditioning factor rasters were normalized to a range of [0, 1] using min–max scaling prior to model input, while categorical layers (lithology, land cover) were encoded numerically using label encoding, assigning a unique integer to each class prior to min–max normalization. These inputs feed into a CNN model for training and evaluation. The model then generates a flood susceptibility map, classifying areas into susceptible and non-susceptible zones, providing critical information for decision-making and risk reduction strategies.

#### Convolutional neural network (CNN)

Convolutional Neural Networks (CNNs) are a class of deep learning models that excel at identifying spatial patterns and hierarchical features in image data^39^. They are effective for tasks where both local detail and broader spatial context are important, making them well-suited for geospatial applications^40^. In this study, a CNN was employed to predict the probability of flood occurrence based on geo-environmental conditioning factors. The CNN is composed of several specialized layers, each serving a distinct purpose in feature learning. The convolutional layers are the core building blocks, applying filters across the input to automatically extract spatial features. These are followed by pooling layers, which reduce the dimensionality of feature maps, retaining the most important information while lowering computational cost and improving model generalization. A global average pooling layer aggregates spatial features into a compact representation, enabling the model to focus on the presence of features rather than their exact position. Finally, full connected (dense) layers integrate the learned features and feed into the output layer to produce the probability of flood occurrence^41^.

The summary of the CNN configuration used in this study is presented in Table 2. The architecture was designed to extract hierarchical spatial features from 64 × 64 × 12 input patches representing the twelve flood conditioning factors. The model consisted of two convolutional blocks with 32 filters and ReLU activation functions, each followed by batch normalization and max-pooling layers for dimensionality reduction and training stability. A global average pooling layer and a dense layer with dropout regularization were used to improve generalization and reduce overfitting. The final sigmoid output layer generated flood susceptibility probabilities ranging from 0 to 1.

**Table 2:** Summary of the convolutional neural network (CNN) architecture used for flood susceptibility mapping, including layer configuration, activation functions, kernel size, stride, and padding parameters.

Layer	Output shape	Parameters	Activation	Kernel/Pool Size	Stride	Padding
Input layer	64 × 64 × 12	–	–	–	–	–
Conv2D-1	64 × 64 × 32	32 filters	ReLU	3 × 3	1	Same
Batch normalization	64 × 64 × 32	–	–	–	–	–
Max pooling-1	32 × 32 × 32	–	–	2 × 2	2	Valid
Conv2D-2	32 × 32 × 32	32 filters	ReLU	3 × 3	1	Same
Batch normalization	32 × 32 × 32	–	–	–	–	–
Max pooling-2	16 × 16 × 32	–	–	2 × 2	2	Valid
Global average pooling	32	–	–	–	–	–
Dense layer	64	64 neurons	ReLU	–	–	–
Dropout	64	Rate = 0.3	–	–	–	–
Output layer	1	1 neuron	Sigmoid	–	–	–

#### Model performance and validation

The development of the CNN model for flood mapping includes data preparation and preprocessing, patch-based sample generation, CNN model development, training and validation using cross-validation, and the generation of the final flood susceptibility map. All spatial data preprocessing, analysis, and CNN modelling were conducted in Python 3.10. Spatial data handling and raster processing were performed using libraries including GeoPandas, Rasterio, NumPy, and Pandas, while visualization and statistical analyses were supported by Matplotlib and Scikit-learn. The CNN model was implemented using TensorFlow 2.12 and Keras. The analysis began with the preparation of input datasets, including a flood inventory and conditioning factors. The flood inventory was provided as a binary raster, where pixels with a value of one denoted known flood and those with zero denoted non-flooded areas. The conditioning factors, organized into a multi-band raster stack. To ensure spatial consistency across datasets, all rasters were aligned to a common reference grid defined by the factor stack. The flood inventory and boundary mask were resampled to match the projection, resolution, and extent of the conditioning factors. Nearest-neighbor interpolation was applied to preserve categorical data integrity. Each factor band was then normalized to a range between zero and one using min–max scaling.

The CNN does not operate directly on individual pixels but instead on patches that capture both local and surrounding contextual information. For this study, 64 × 64-pixel patches were extracted from the factor stack. The 64 × 64 pixel patch size was selected based on the spatial scale of flood-relevant geomorphological features in the study area, capturing approximately 1.9 km × 1.9 km of landscape context at 30 m resolution. To prevent spatial data leakage, training and validation patches were required to have non-overlapping spatial footprints, with a minimum center-to-center distance of 64 pixels (1,920 m) between any training and validation sample. Balanced sampling was critical given the natural class imbalance between flooded and non-flooded areas; therefore, an equal number of positive (flooded) and negative (non-flooded) samples were randomly selected, with a maximum of 500 patches per class. The 500-patch-per-class ceiling was determined empirically: increasing the sample size to 1,000 patches per class yielded no statistically significant improvement in Area Under the Receiver Operating Characteristic Curve (AUC).

The CNN was trained using the Adam optimizer and binary cross-entropy loss^42,43^, which is appropriate for binary classification problems. Training was conducted with a batch size of 64 for a maximum of 40 epochs. To prevent overfitting, two strategies were applied: early stopping, which halted training when validation performance plateaued, and learning rate reduction, which adjusted optimizer updates for finer convergence. The AUC was calculated to evaluate the model’s discriminative power across different threshold levels. Accuracy and loss were tracked during training, while precision, recall, F1-score, and AUC provided comprehensive insight into classification performance. The final susceptibility map was produced by applying the trained CNN across the entire study area using a sliding window approach. For each valid pixel, a patch centered on that location was extracted, and the CNN generated a probability score between 0 (no flood) and 1 (high likelihood of flood). A five-fold cross-validation strategy was implemented to assess generalizability. The dataset was partitioned into five equal subsets, where each fold was used once for validation and four times for training. Model performance was evaluated using multiple metrics (Eqs. 1–4) derived from the confusion matrix as

1 $$\mathrm{A}\mathrm{c}\mathrm{c}\mathrm{u}\mathrm{r}\mathrm{a}\mathrm{c}\mathrm{y}=\frac{\mathrm{T}\mathrm{P}+\mathrm{T}\mathrm{N}}{\sum \mathrm{T}\mathrm{P}+\mathrm{F}\mathrm{P}+\mathrm{T}\mathrm{N}+\mathrm{F}\mathrm{N}}$$

2 $$\mathrm{P}\mathrm{r}\mathrm{e}\mathrm{c}\mathrm{i}\mathrm{s}\mathrm{i}\mathrm{o}\mathrm{n}=\frac{\mathrm{T}\mathrm{P}}{\mathrm{T}\mathrm{P}+\mathrm{F}\mathrm{P}}$$

3 $$\mathrm{R}\mathrm{e}\mathrm{c}\mathrm{a}\mathrm{l}\mathrm{l}=\frac{\mathrm{T}\mathrm{P}}{\mathrm{T}\mathrm{P}+\mathrm{F}\mathrm{N}}$$

4 $$F1Score=\frac{2 \times \mathrm{P}\mathrm{r}\mathrm{e}\mathrm{c}\mathrm{i}\mathrm{s}\mathrm{i}\mathrm{o}\mathrm{n}\times \mathrm{R}\mathrm{e}\mathrm{c}\mathrm{a}\mathrm{l}\mathrm{l}}{\mathrm{P}\mathrm{r}\mathrm{e}\mathrm{c}\mathrm{i}\mathrm{s}\mathrm{i}\mathrm{o}\mathrm{n}+\mathrm{R}\mathrm{e}\mathrm{c}\mathrm{a}\mathrm{l}\mathrm{l}}$$

where TP is the correctly predicted flood samples, TN is the correctly predicted non-flood samples, FP non-flood samples misclassified as floods, and FN are the flood samples misclassified as non-floods.

#### Feature importance analysis

To interpret the CNN model and quantify the relative contribution of each conditioning factor to the final prediction, a permutation feature importance analysis was conducted. This model-agnostic method works by randomly shuffling the values of a single feature in the validation dataset and measuring the resulting decrease in the model’s performance metric (in this case, the F1-Score). A large decrease in performance indicates that the model relied heavily on that feature for accurate predictions. The importance score for each factor was calculated as the normalized decrease in the F1-Score after permutation, resulting in the percentage contributions. This approach provides a robust and intuitive measure of feature relevance.

## Results and discussion

### Conditioning factors

**Fig. 3:**
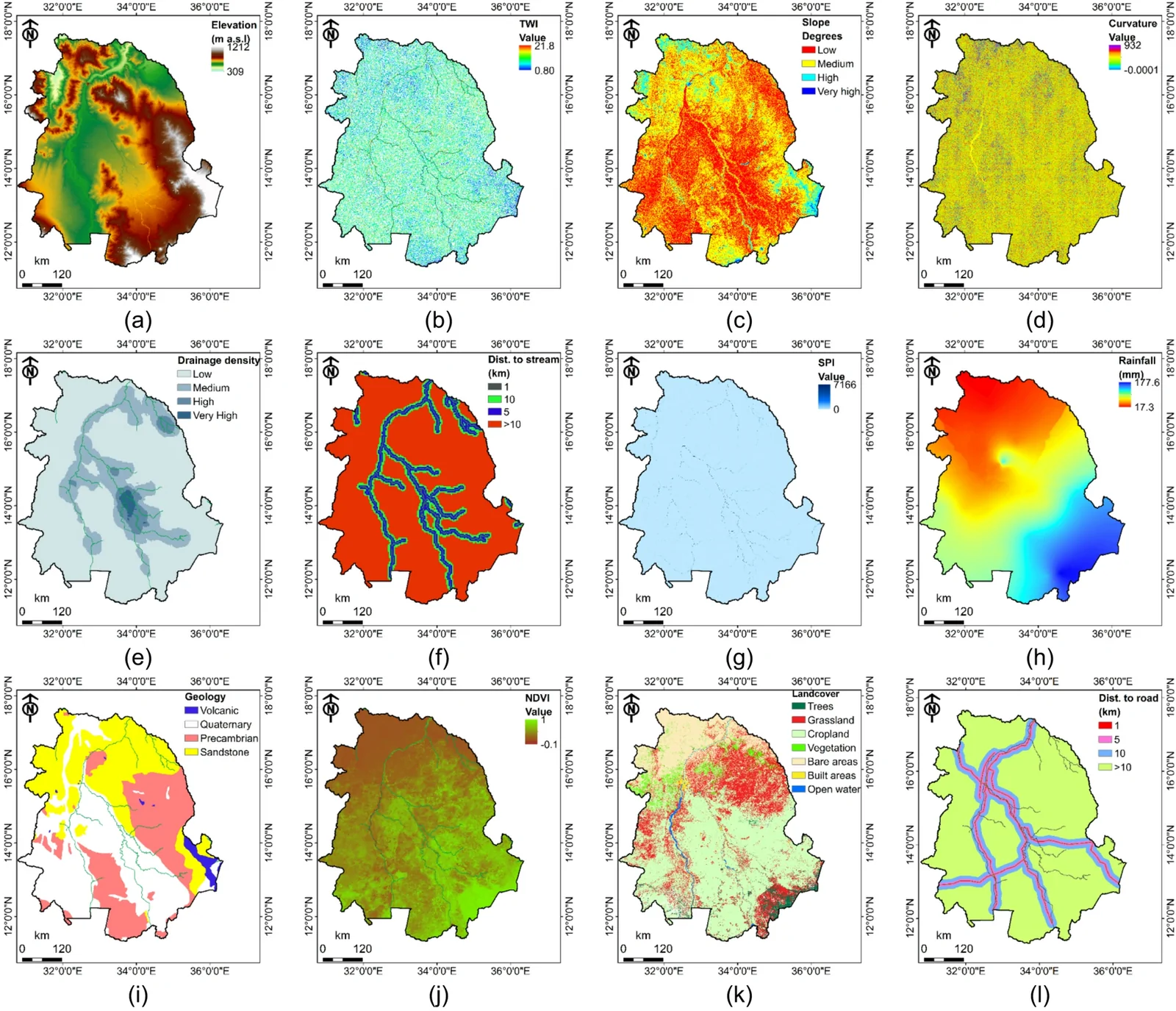
Flood conditioning factors used for CNN-based flood susceptibility mapping, including topographical factors: (**a**) elevation, (**b**) topographic wetness index (TWI), (**c**) slope, and (**d**) curvature; hydrological factors: (**e**) drainage density, (**f**) distance to stream, (**g**) stream power index (SPI), and (**h**) rainfall; and geological and anthropogenic factors: (**i**) geology, (**j**) normalized difference vegetation index (NDVI), (**k**) land cover, and (**l**) distance to roads.

The elevation map (Fig. 3a) shows that low-lying alluvial plains along the Blue Nile, White Nile, and main Nile constitute the most flood-prone areas due to their role as natural floodwater accumulation zones during high-flow periods. The Topographic Wetness Index (TWI) map (Fig. 3b) highlights valley bottoms and topographic depressions with high moisture accumulation and reduced infiltration capacity, indicating increased flood susceptibility. The slope map (Fig. 3c) reveals that the flat to gently sloping terrain in Jazirah and Sennar states favors prolonged inundation and poor drainage, whereas steeper eastern highlands promote rapid runoff generation and localized flash flooding. The curvature map (Fig. 3d) further identifies concave terrain zones where runoff converges and floodwater accumulates.

The drainage density map (Fig. 3e) indicates that areas with dense stream networks, particularly along the Blue Nile and White Nile corridors, are highly susceptible to flooding due to rapid hydrological response. Similarly, the distance-to-stream map (Fig. 3f) shows that areas located within 1–3 km of major rivers experience the highest riverine flood risk from overbank flow and channel overflow. The Stream Power Index (SPI) map (Fig. 3g) identifies steep channel reaches with greater erosive and flash flood potential, while the rainfall map (Fig. 3h) demonstrates that the southeastern region receives higher precipitation, contributing to increased runoff generation and flood initiation.

The geology map (Fig. 3i) indicates that Quaternary alluvial deposits along river corridors are highly susceptible to inundation and waterlogging, whereas impermeable Precambrian and volcanic rocks enhance surface runoff. The NDVI map (Fig. 3j) shows that sparsely vegetated and bare areas exhibit reduced infiltration and higher flood susceptibility, while vegetated regions improve infiltration and reduce runoff. The land cover map (Fig. 3k) further reveals that built-up and bare surfaces are associated with higher flood risk due to low permeability, whereas vegetated and agricultural areas provide greater hydrological buffering. Finally, the distance-to-roads map (Fig. 3l) highlights that areas close to roads are more flood-prone because compacted surfaces disrupt natural drainage and increase surface runoff.

### Correlation between conditioning factors

**Fig. 4:**
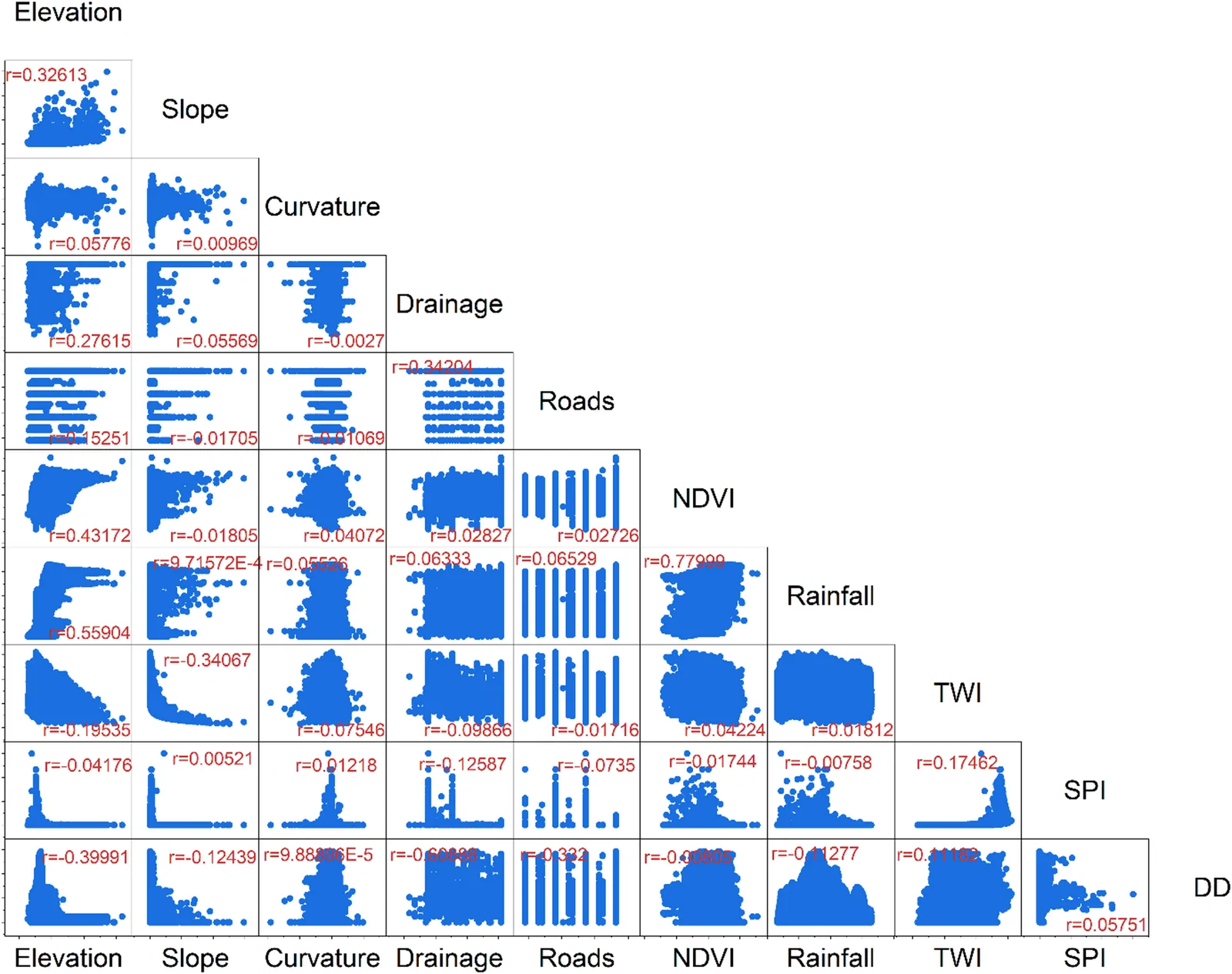
Scatter matrix showing correlation between different conditioning factors using Pearson coefficient.

A Pearson correlation (r) analysis was conducted to evaluate the relationships among the flood conditioning factors and assess potential multicollinearity (Fig. 4). Most factor pairs exhibited weak to moderate correlations (generally r < 0.6), indicating limited redundancy among variables. The strongest positive correlation was observed between NDVI and rainfall (r = 0.78), reflecting the influence of precipitation on vegetation density, while elevation and rainfall showed a moderate correlation (r = 0.56) associated with orographic rainfall effects in the eastern highlands. Several factors exhibited negligible correlations, such as curvature–drainage density (r =  − 0.003) and SPI–elevation (r =  − 0.04), indicating that these variables capture distinct geomorphological and hydrological characteristics. The absence of severe multicollinearity suggests that the selected conditioning factors provide complementary environmental information for flood susceptibility modelling^28^. Although CNN models are inherently more robust to correlated predictors than traditional statistical models, correlation analysis remains important for identifying highly redundant variables that may introduce unnecessary complexity into the model^44^. The results therefore support the suitability of the selected factors for CNN-based modelling of complex, non-linear flood processes and confirm that each variable contributes meaningful and partially independent information to the susceptibility assessment.

### CNN-based modelling

**Fig. 5:**
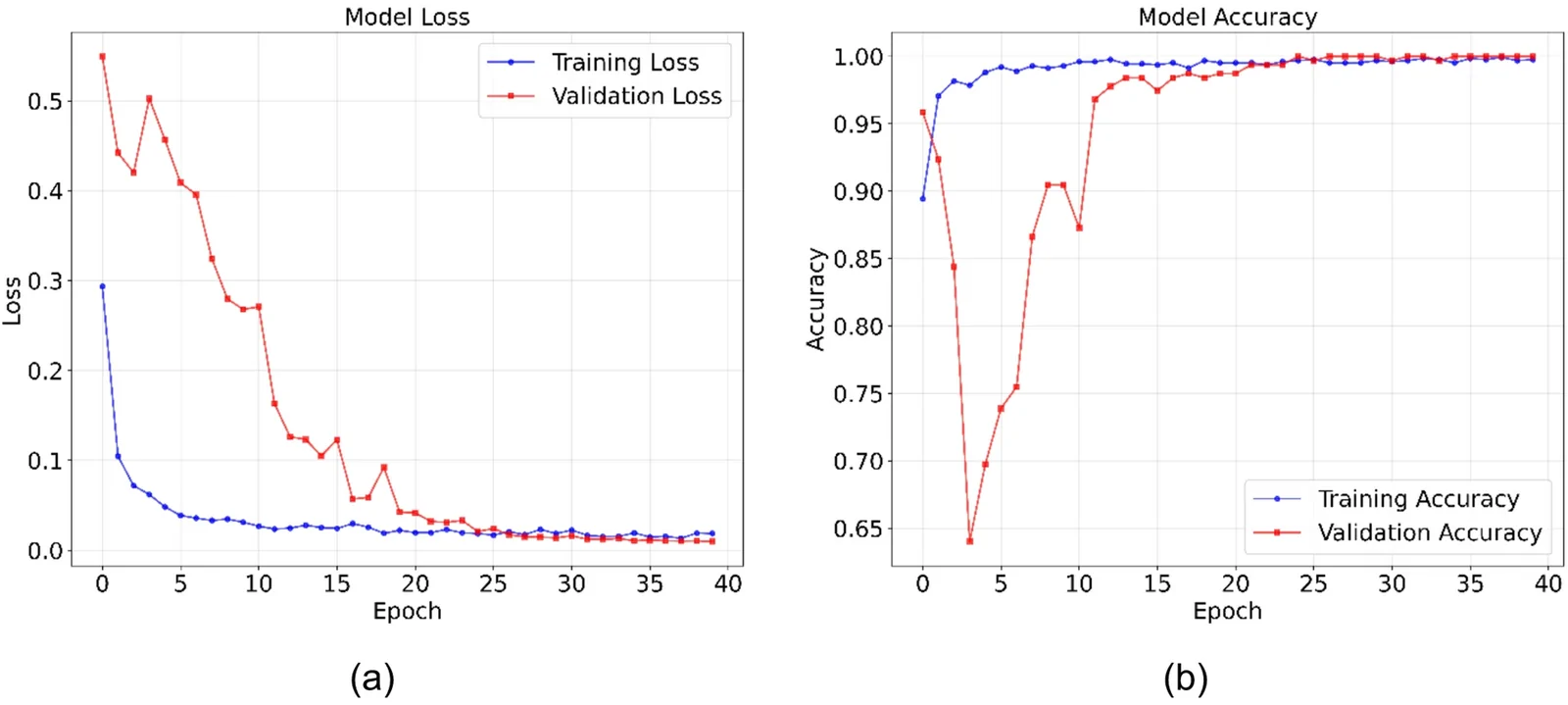
The representation of training and validation using (**a**) binary cross-entropy loss function and (**b**) accuracy metric.

The CNN is used to map the flood susceptibility with the non-correlated conditioning factors as input and the flood inventory map as output. The model exhibited good performance in training and validating flood susceptibility across the study area. The model was trained using the binary cross-entropy loss function, with accuracy employed as an additional performance indicator. As presented in Fig. 5a, the training loss rapidly decreased during the first few epochs, showing a steep decline from approximately 0.55 to below 0.05, while the validation loss followed a similar trend with minor fluctuations before stabilizing around a comparably low value after roughly 20 epochs. The corresponding accuracy curves shown in Fig. 5b reinforce this observation. The model’s training accuracy quickly improved, exceeding 0.95 after only a few epochs. The convergence of training and validation accuracy curves reflects the robust learning capability of model and its generalization strength in distinguishing between flooded and non-flooded areas.

The CNN model demonstrated strong predictive performance, achieving high classification metrics on the validation dataset (AUC = 0.974 and F1-score = 0.96). These results indicate that the model effectively captured the complex relationships between flood occurrence and the selected conditioning factors while maintaining good generalization capability. Similar levels of performance have been reported in recent CNN-based flood susceptibility studies, where deep learning models successfully identified non-linear spatial patterns from heterogeneous environmental datasets^45^. The strong predictive capability observed in this study reflects the pronounced geomorphological and hydrological contrasts within the study area, particularly between the low-lying alluvial floodplains and the elevated arid terrains surrounding the Blue Nile, White Nile, and main Nile corridors.

The model effectively learned the spatial characteristics associated with flood-prone environments, including low elevation, gentle slopes, high TWI values, and proximity to major river channels. The use of five-fold cross-validation, combined with dropout and batch normalization, helped reduce overfitting and improved model robustness across different spatial subsets. Nevertheless, the influence of spatial autocorrelation cannot be fully excluded, as neighboring samples may still share similar environmental characteristics^46^. Therefore, future studies should incorporate temporally independent flood events and spatial blocking strategies to further assess model transferability and predictive reliability.

**Fig. 6:**
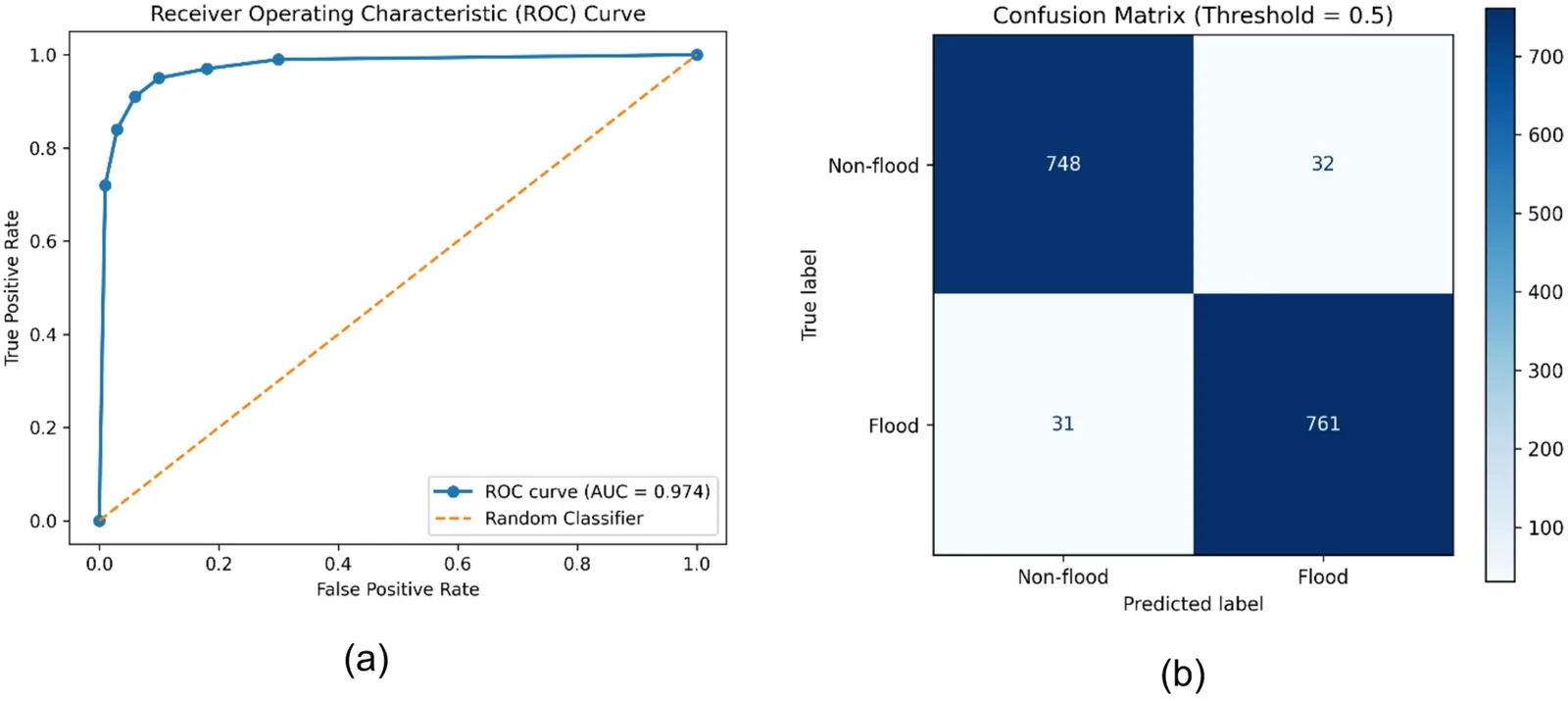
The performance of the CNN model based on the (**a**) ROC curve and (**b**) confusion matrix.

The discrimination ability of the CNN model is illustrated by the ROC curve presented in Fig. 6a. The curve remains close to the upper-left corner of the plot, corresponding to an AUC value of 0.974, which indicates excellent discrimination between flood and non-flood classes. Although a small number of misclassifications occurred, the model maintained high sensitivity and specificity across classification thresholds. The confusion matrix (Fig. 6b) further demonstrates the classification performance, where most flood and non-flood samples were correctly identified. The model produced 748 true negatives and 761 true positives, with 32 false positives and 31 false negatives, resulting in high precision, recall, and overall classification accuracy.

The convergence of the training and validation loss curves, ideal ROC curve, and flawless confusion matrix demonstrates the exceptional predictive capability of the CNN model in mapping flood susceptibility. This performance indicates that the CNN effectively learned complex, non-linear spatial relationships among conditioning factors, enabling it to accurately distinguish flood-prone from safe zones. Similar findings have been reported in previous studies where deep learning models, particularly CNNs, outperformed traditional statistical and machine learning approaches such as logistic regression, random forest, and support vector machines^44,47,48^. The superior accuracy of CNNs arises from their ability to automatically extract hierarchical spatial features, capture local dependencies, and model intricate interactions among conditioning factors^49^. In comparison, studies applying conventional methods like frequency ratio often reported moderate accuracy and sensitivity to multicollinearity^50,51^, as these approaches assume linear relationships between conditioning factors and flood occurrence. Ensemble models such as random forest and gradient boosting^26^ have shown improved robustness but still rely on manually engineered features. In contrast, the CNN in this study autonomously identified relevant spatial features from input data and integrated them into an optimized predictive structure. These results confirm that CNN is a highly suitable and effective choice for flood susceptibility mapping in these complex riverine environments.

### Flood susceptibility map

**Fig. 7:**
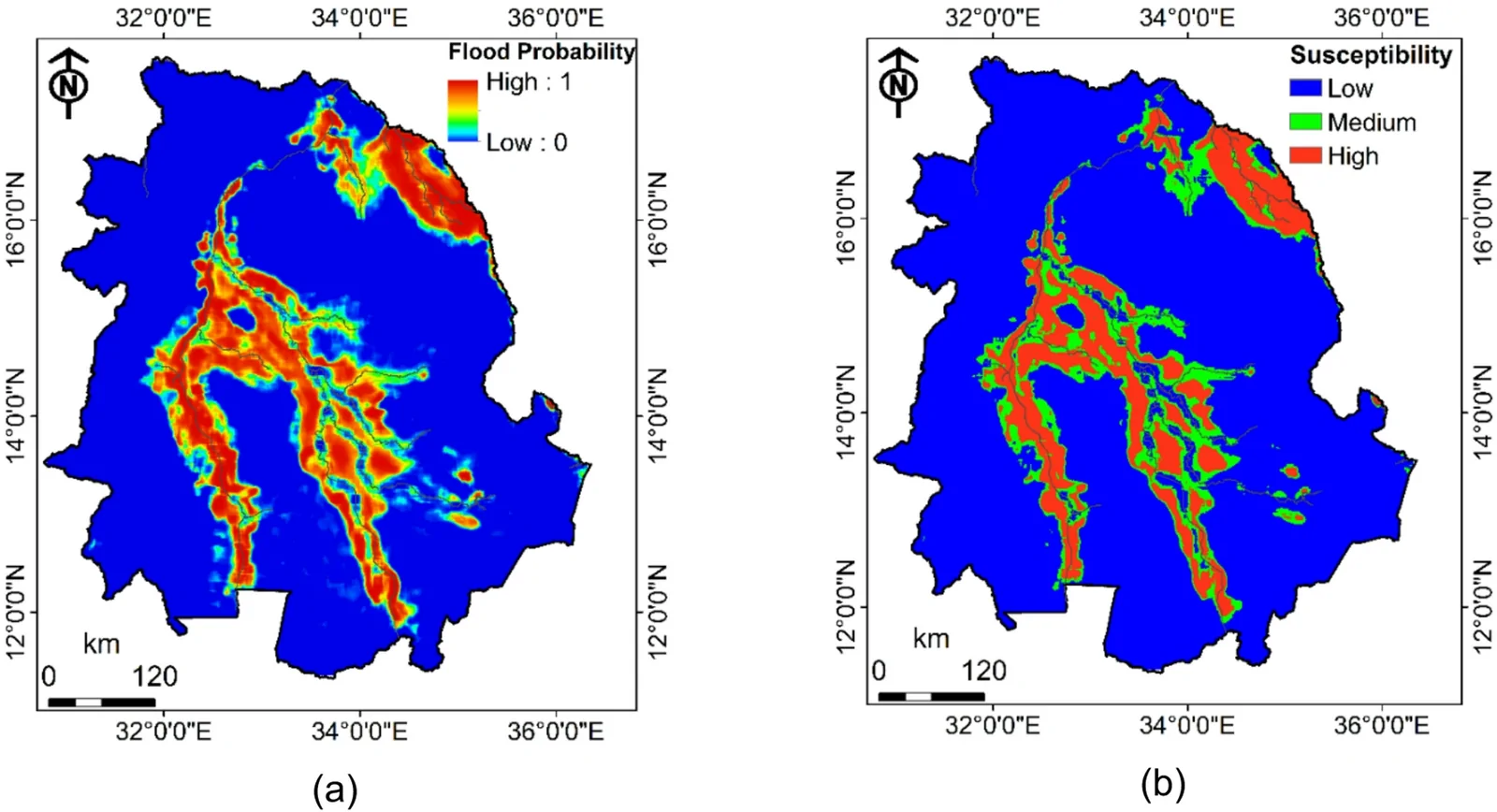
(**a**) Flood probability and (**b**) flood susceptibility maps generated using convolutional neural networks.

The integration of all topographical, hydrological, geological, and anthropogenic conditioning factors into the CNN model revealed the nature of flood risk across the study area. The flood susceptibility assessment generated a continuous probability map (Fig. 7a) and a categorical classification map (Fig. 7b). The continuous probability map showed the flood susceptibility as a gradient of risk, with probability values ranging from a minimum of 0 (no susceptibility) to 1 (absolute susceptibility). Most of the study area is dominated by very low flood probability, representing elevated terrain and areas distant from major watercourses. The transition zones delineate the progressive increase in flood probability that closely follow the Blue Nile, White Nile, and main Nile River networks. The highest probability values form distinct corridors along the immediate riparian zones of all three rivers, with particularly extensive high-probability areas concentrated in two critical regions: the Khartoum confluence zone, where the Blue and White Nile merge, and the central agricultural plains of Jazirah and Sennar states. These zones are associated with multiple high-risk conditioning factors, including the lowest elevations, flattest terrain, highest TWI values, and immediate proximity to rivers (distance < 1 km).

The continuous probability output (0–1) was classified into three susceptibility zones using the following thresholds: Low susceptibility (probability < 0.30) encompasses areas where model-predicted flood probability is below the threshold of hydrological significance, typically corresponding to elevated, well-drained terrain. Medium susceptibility (0.30–0.65) delineates transitional zones subject to flooding during above-average rainfall or extreme events. High susceptibility (> 0.65) identifies areas at substantial risk from seasonal riverine flooding. These thresholds were determined through an analysis of the bimodal probability distribution of model outputs and cross-referenced with the spatial distribution of historical flood polygons to ensure physical consistency. The medium susceptibility class forms transitional zones between low-risk interior areas and high-risk riparian corridors, located 3–10 km from major river channels where flood influence diminishes but remains present through tributary connections. These medium-risk areas represent important buffer zones that may experience flooding during extreme events but generally remain dry during typical seasonal flood cycles. The high susceptibility class delineates areas with substantial flood risk, located 1–5 km from major rivers in the floodplain. These areas form continuous corridors closely following the Blue Nile, White Nile, and main Nile channels, with widths ranging from several hundred meters in confined valley sections to several kilometers in broad alluvial plains.

### Contribution of factors to flood map

**Fig. 8:**
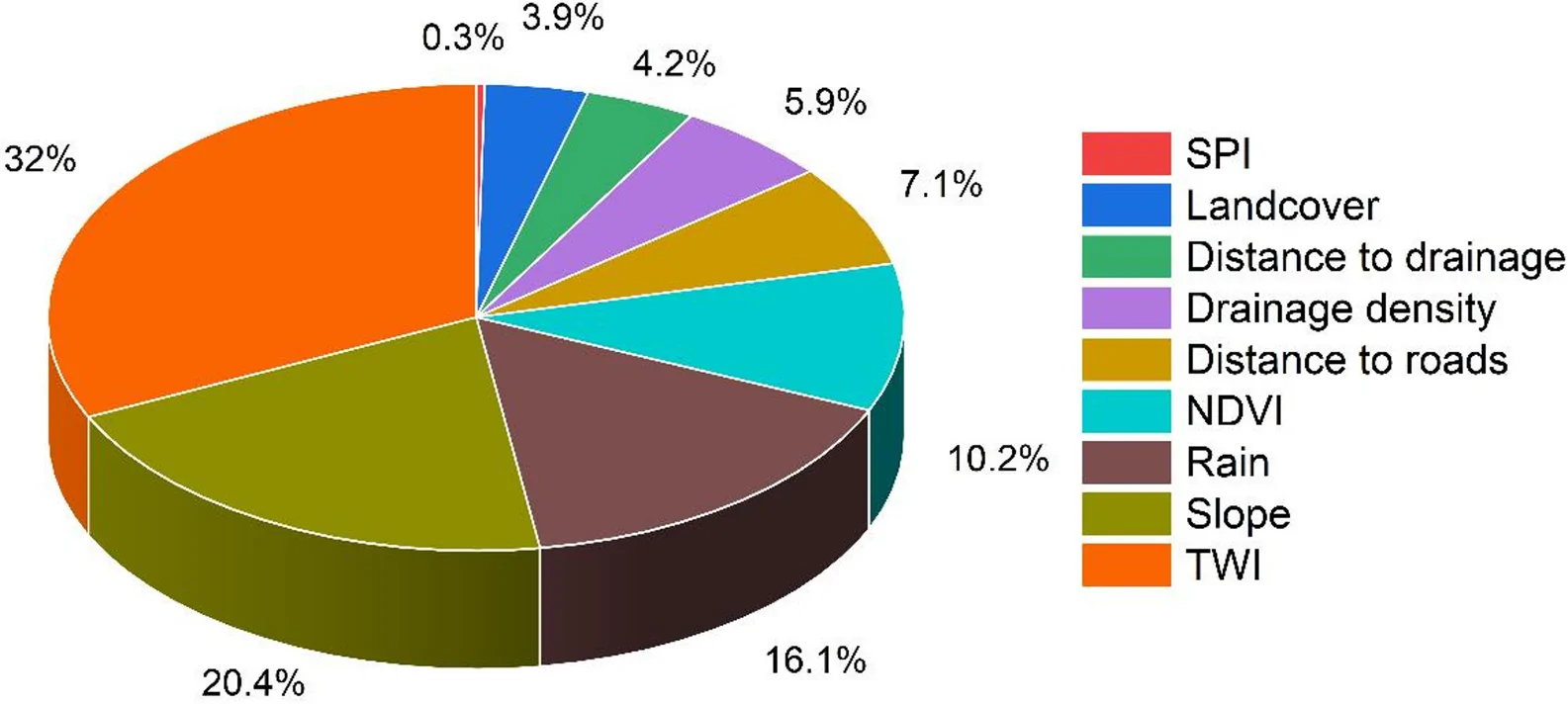
Pie chart showing the contribution of different conditioning factors to the flood susceptibility map.

Analysis of the feature importance quantified the relative contribution of each conditioning factor to the flood susceptibility (Fig. 8). TWI emerged as the most influential factor (32%), reflecting its role in combining upslope contributing area and slope gradient to capture water accumulation potential. Slope ranked second (20.4%), confirming that terrain steepness critically controls flow velocity and runoff generation, while steeper terrains promote rapid drainage. The primacy of TWI and slope in the present study is consistent with findings from analogous CNN-based flood susceptibility studies. For example, Feizbahr et. al^52^ reported TWI as the dominant factor in Texas, United States, while Rahman et. al^53^ found that slope is explaining most of model variance in riverine flood susceptibility mapping. Rainfall (16.1%) ranked third, validating its role as the primary hydrological input; however, its lower importance compared to TWI and slope suggests that where water accumulates is more decisive than how much rainfall occurs in this large riverine system^54^. Among moderately important factors, NDVI (10.2%) reflects the influence of vegetation cover on infiltration, thereby reducing flood susceptibility in vegetated areas. Distance to roads (7.1%) and drainage density (5.9%) indicate the hydrological and anthropogenic effects of infrastructure and drainage patterns. SPI showed negligible importance (0.3%), confirming its limited relevance for inundation mapping compared to erosion dynamics.

Elevation, curvature, and geology showed low contribution, likely due to redundancy with derivative variables and coarse classification resolution. Elevation’s influence is inherently embedded within TWI and slope computations, while geological variations may indirectly manifest through vegetation and surface moisture patterns captured by NDVI and TWI. In general, feature weighting aligns closely with hydrological principles, demonstrating that flood susceptibility in the Blue Nile, White Nile, and main Nile corridors is primarily controlled by terrain-driven water accumulation and flow regulation. The distributed importance across multiple variables (no single factor exceeding 32%) confirms that flood susceptibility is inherently multi-factorial, reinforcing the advantage of the CNN models in capturing complex, non-linear interactions beyond the scope of traditional linear or overlay-based approaches. However, it is important to note that permutation feature importance has several limitations, including potential underestimation of feature contributions, and its inability to explain causal relationships or internal CNN feature representations. Therefore, future studies could incorporate advanced interpretability techniques such as SHAP or Grad-CAM for deeper analysis of CNN decision-making processes.

### Implications and recommendations

The flood susceptibility assessment revealed vulnerabilities with profound implications for urban planning, agriculture, and sustainable water resources management. In urban areas, particularly the Khartoum metropolitan area, very high susceptibility zones encompass extensive built-up areas and low-lying alluvial terrain, exposing residential neighborhoods, government institutions, and critical infrastructure to severe flood risks, as demonstrated during the 2020 floods^35^. To mitigate these risks, the study recommends establishing risk-based zoning, designating very high susceptibility zones for flood-compatible uses such as natural floodplains, and imposing stringent controls on residential and commercial development in high-risk areas.

Most productive agricultural areas, including Khartoum and the Gezira irrigation scheme, fall largely within high susceptibility zones^55^. Agricultural planning should adopt spatially differentiated crop management, prioritizing flood-tolerant and short-duration crops in high-risk areas and improving field drainage. Protecting irrigation infrastructure requires elevation of critical nodes, canal scour protection, and redundancy in water delivery systems, while on-farm drainage and retention basins should be systematically implemented. Dam operations at Roseires, Sennar, and the Grand Ethiopian Renaissance Dam should adopt risk-based strategies, pre-releasing water during high-risk periods, coordinating releases to desynchronize peak flows, and sharing real-time hydrological data^36^.

## Conclusions

Sudan experiences devastating seasonal floods every fall that claim lives, destroy infrastructure, displace populations, and disrupt agricultural production. This study addressed the flood susceptibility assessment by developing a CNN deep learning model that integrates twelve topographical, hydrological, geological, and anthropogenic conditioning factors in central-eastern Sudan. The conclusions of the study can be summarized as follows:

- The CNN model demonstrated high predictive performance, successfully delineating three susceptibility classes that reveal very high flood risk concentrated along immediate river corridors, at the Khartoum metropolitan confluence area, and low-lying Quaternary alluvial plains in Jazirah and Sennar states.
- The analysis confirmed that flood susceptibility results from complex interactions among multiple factors, with maximum vulnerability occurring where high TWI values, proximity to major rivers, low vegetation cover, and intensive urbanization converge.
- The study demonstrates the effectiveness of CNN-based flood susceptibility mapping in a large-scale, data-constrained semi-arid environment, providing a reproducible and spatially explicit framework for central-eastern Sudan that can be transferred to other data-scarce riverine regions within the Nile Basin and across Africa.

Despite these contributions, the study has several limitations that should be considered. First, the model is a static representation and does not capture the dynamic, temporal nature of flood events, such as variations in intensity, duration, or antecedent soil moisture. Second, the susceptibility map indicates ‘where’ flooding is likely but not ‘how’ it manifests; coupling this approach with hydraulic models (e.g., HEC-RAS) would provide crucial information on flood depth and velocity. Future research should incorporate socioeconomic vulnerability indicators to create a comprehensive flood risk assessment and assess the impacts of climate change on future flood patterns and susceptibility.

## Data Availability

The data that supports the findings of this study are available from the corresponding author upon reasonable request.
